# Stigma as a barrier and sex work as a protective factor for HIV testing among trans women in Nepal

**DOI:** 10.1371/journal.pgph.0001098

**Published:** 2023-03-17

**Authors:** Erin C. Wilson, Caitlin M. Turner, Manisha Dhakal, Sanjay Sharma, Anuj Rai, Rajesh Lama, Swagata Banik, Sean Arayasirikul

**Affiliations:** 1 San Francisco Department of Public Health, Trans Research Unit for Equity (TRUE), Center for Public Health Research, San Francisco, California, United States of America; 2 Blue Diamond Society, Kathmandu, Nepal; 3 Department of Public Health & Prevention Sciences, Baldwin Wallace University, Berea, Ohio, United States of America; National University of Singapore, SINGAPORE

## Abstract

Stigma towards trans women in Nepal creates individual and system-level risks for HIV. A critical protective factor is access to HIV prevention. Research is needed to determine the impact of stigma on HIV testing among trans women in Nepal. We conducted a secondary analysis of data collected using respondent driven sampling in 2019 on HIV risk among trans women in Nepal. Data analysis was restricted to trans women who were HIV negative at testing through the parent study. Descriptive statistics, tests for bivariable associations between HIV testing and stigma variables, and binomial Poisson regression were conducted to examine HIV testing outcomes. There were 173 participants who tested negative for HIV in our sample. The majority were under age 35 (59%) and most had a grade school education or less (64.7%). No trans women were homeless and most rented a room (70.5%) or owned their home (19.7%). The majority were currently sex workers (57.8%). Almost all HIV-negative trans women had ever been tested for HIV (90.8%), but only 53.5% in the last 3 months. The most frequently cited reason for not having been tested was thinking they were at low risk for HIV (40.9%) and being afraid of receiving a positive test result (22.7%). HIV and anti-trans stigma were high across most measures, including that almost all (94.2%) believed that most people in Nepal would discriminate against people with HIV. And most participants thought trans women were not accepted in Nepali Society (65.9%). Most participants also reported high social support (70.5%). Social cohesion among participants varied, with most experiencing medium (41.6%) or high (33.5%) social cohesion. Just over half had high social participation (55.5%). Participants who reported current sex work had lower prevalence of not testing for HIV in the last 3 months (prevalence ratio, PR = 0.54, 95% confidence interval, 95%CI = 0.32–0.92, p = 0.02). Every one-unit increase in social cohesion was associated with 1.05 times the prevalence of not testing for HIV in the last 3 months (95%CI = 1.01–1.09, p-value = 0.02). Trans women who did sex work were more likely to be HIV tested while those who were more socially connected to peers were less likely to have recently been tested for HIV. HIV stigma may result in fear of social rejection from peers if one tests positive. Interventions that focus on addressing stigma within trans women’s social networks and strategies to mitigate HIV stigma in society may result in increased frequency of HIV testing among trans women in Nepal.

## Introduction

In low- and middle-income countries, trans women have 77.5 times greater odds of having HIV compared to cisgender women [1]. Nepal is a low-income country in South Asia with a concentrated HIV epidemic among key populations, including trans women [2]. In 2019,11.3% of trans women in Kathmandu, Nepal was estimated to be living with HIV, which is twice the prevalence found in prior studies and higher than in all other key populations except for people who inject drugs [3].

Globally, one of the biggest barriers to ending the HIV epidemic is HIV testing coverage and frequency [4]. Estimates suggest that less than half of people living with HIV in low- and middle-income countries (LMICs) regions know their HIV status [5]. HIV status awareness in South Asia ranges widely from Thailand where almost all people living HIV know their status to only 26% in Indonesia [6]. Research on HIV testing among trans women in LMICs is limited, but a recent study found that only 49.2% of trans women in a national survey in Cambodia had been tested for HIV in the last 6 months [7].

HIV risk for trans women in Nepal and engagement in HIV prevention is highly influenced by the stigmas they face [8]. Nepal has a conservative patriarchal society highly valuing masculinity over femininity [9]. Stigma for trans women in Nepal is rooted in the perception that they do not conform to masculine Nepali gender norms [10]. Strict cisgender heterosexual norms drive discrimination and violence in many sectors of Nepali society [11,12]. In prior analyses, we found high internalized, anticipated, and experienced stigma among trans women [13]. For example, more 87.86% of Nepali trans women hid their gender identity, 71.20% felt unaccepted in Nepali society, and 55.34% reported being arrested because they were trans. The ways in which stigma operates in systems may present barriers to HIV testing for trans women in Nepal. For example, trans women may avoid healthcare settings and HIV testing due to anticipated provider stigma based on prior discriminatory behavior [14]. Structural barriers that reduce access to education may lead to lower HIV knowledge and risk perception, presenting barriers to HIV testing [15,16]. Furthermore, trans women face intersectional discrimination from the interaction between HIV stigma and anti-trans stigma, which together foster unique experiences of oppression that present additional structural barriers to HIV testing [17]. For example, almost 100% of HIV infections in a recent study of trans women in Vietnam were unrecognized because trans women did not want to affirm stereotypes related to HIV among trans women [18].

Factors that are protective of the negative impacts from stigmas may reduce barriers to HIV testing. Social cohesion, operationalized as bonding with community members, can mitigate stigma among marginalized populations [19]. For trans women in Nepal, building social cohesion may be a challenge because many trans women cannot be visible during the day due to the threat of violence and persecution [10,20]. Social participation, or bridging with those outside the trans community, may provide a buffer from stigma and help promote health seeking behaviors. For example, an intervention with sex workers in Swaziland found that social cohesion and social participation were significantly associated with health seeking behaviors like HIV testing [21]. Available evidence suggests that at the most basic level, social participation can increase feelings of safety for HIV status disclosure that are central to seeking HIV prevention services [19]. Yet again, social participation for trans women may be difficult due to family and societal rejection [10,22]. Finally, social support is another factor that may reduce internalized negative beliefs and thus encourage health seeking behaviors [23].

The purpose of this analysis is to identify risk and protective factors associated with HIV testing among trans women not living with HIV in Nepal. The goal of this analysis was to provide evidence-based directions for interventions that address stigmas-related barriers to HIV testing that trans women in Nepal face.

## Methods

### Ethical approval

The protocol was reviewed by the University of California, San Francisco Human Research Protection Program and given expedited review approval with designation of minimal risk research. The Baldwin Wallace University Human Subjects Review Committee and the Nepal Health Research Council of the government of Nepal also approved the research protocol. Written informed consent was obtained from participants. Consent was documented on a paper consent.

### Data collection

*Sweekar* (“acceptance” in Nepali*)*, was a population-based bio-behavioral study conducted in collaboration with the Blue Diamond Society (BDS) in 2019. Blue Diamond Society is the vanguard non-governmental organization working throughout Nepal for sexual and gender minority equal rights, economic empowerment, livelihood support, representation, and protection. Methods for the study are described in depth elsewhere [13], but briefly, we used respondent-driven sampling to recruit a diverse sample of 200 trans women participants from Kathmandu, Nepal between May and October of 2019. Eligible participants were 18 years old or older, identified as transgender, hijra, Meti, third gender (or Tesoro lingi, which means assigned male at birth and legally recognized as other category) or anything other than a gender typically associated with their male sex at birth, lived in the Kathmandu Valley and spoke Nepalese or English.

Trained staff who self-identified as trans or were part of the lesbian, gay, bisexual, and transgender community (LGBT) conducted survey data collection, HIV testing, counseling and provided referrals to participants. Surveys were conducted using an interviewer-administered electronic questionnaire. The survey was translated from English into Nepali and offered in both languages. Most survey interviews were conducted in the native tongue of Nepali. Seeds and subsequent recruiters received an incentive comparable to USD$10 for participation in the survey and HIV testing and received remuneration for all referrals who enrolled in the study. All participants in this analysis agreed to being tested for HIV. We measured HIV status using rapid HIV testing with whole blood after completion of pre-test counseling by a counselor at BDS. Rapid HIV testing was conducted using a serial testing scheme based on the Nepal national algorithm and using rapid HIV test kits [24]. All participants received post-test counseling, with specific messages tailored to their test result. BDS provides on-site, HIV testing, comprehensive health education, and LGBT-competent resources with trained staff. Persons with a reactive result or indeterminate result were given referrals to HIV care services and further counseling and testing.

### Measures

Measures were developed in close collaboration between the US and Nepal-based teams with incorporation of items to meet the study aims, prior measures used in behavioral surveys in Nepal by BDS and the US team, and with input from community stakeholders. Twenty stakeholders were interviewed from NGOs interested in addressing stigma in health and HIV prevention and care; researchers, academicians, and HIV care providers interested in similar topics; government and civic officials; business and cultural community stakeholders. Leaders from the trans women community, including those from the BDS, were also invited to consult on measures.

#### Socio-demographic factors

Socio-demographic factors were captured to descriptively characterize trans women and their social and economic status in the Nepali context. Trans women were asked about their age, gender identity, education, housing, sex work, employment and income. Sex work was measured as having exchanged sex for money, good or a place to stay. We asked about ever doing sex work in their lifetime and currently doing sex work. For employment, trans women were asked level of employment (e.g., full or part time) for any type for job, including sex work, and they were asked if they were students or retired. Income ranges were categorized based minimum wage requirement and variance within reported income by trans women participants. Nepal’s minimum wage at the time of the study was 8,000 NRS per month, and so we used this as the lowest income category, followed by larger increments of 4,000 NRS. The largest category used was greater than 20,000 NRS per month, which is still the lowest average salary in Nepal, but one of the higher monthly incomes reported by trans women in our study.

#### Healthcare access, HIV testing and HIV knowledge

Healthcare access was measured by asking if participants have medical care in the city of Kathmandu where they reside, and if they have access to gender affirming care. HIV testing behaviors were assessed by asking participants if they had ever been tested before, and if so, where had they been tested. Additionally, participants could indicate whether they were interested in home HIV testing. We also asked when the last time they were tested for HIV was, and we then split these dates into categories of having been tested in the last 3 and 6 months, in accordance with international guidelines suggesting frequent retesting for key populations at high risk for HIV [25]. Participants were given a set of responses and an “Other, specify” option to assess why they had not been tested for HIV recently. This question was only asked of those who had not been tested for HIV in the last 6 months. HIV knowledge (Cronbach’s alpha = 0.91) was assessed with a composite score summing the number of items answered correctly in a brief HIV knowledge questionnaire [26].

#### HIV and anti-trans stigma

We measured two types of stigma that we hypothesized would impact HIV testing behavior. The first was HIV stigma, which was comprised of four questions. The first two were whether most people in Nepal would discriminate against people with HIV or believed that most people in Nepal would think that those with HIV got what they deserved. Participants were also asked if they believed most people in Nepal would support the rights of someone with HIV and whether most people in Nepal would be friends with someone living with HIV. Likert scale responses were binarized as yes for agree or strongly agree and no for disagree or strongly disagree. We also assessed anti-trans stigma with two items. The first was a question about internalized stigma that asked if participants had to hide their identity from family in the last year, and the second was assessing experienced stigma of whether or not participants were ever arrested for being trans, with the following question: “In your lifetime, do you believe you have ever been unfairly arrested for being trans?” We also asked about anti-trans stigma in healthcare settings with the following three experienced stigma questions: “Have you had to educate doctors, nurses, or administrative staff about transgender-related health care?,” “Have you experienced maltreatment in health care settings because of your identity?,” and “Have you experienced mistreatment in the past year because of your gender identity from a clinical staff person?”

#### Social support, cohesion, acceptance, and participation

To measure social support, we used a modified version of the multidimensional scale of perceived social support (Cronbach’s alpha = 0.94) that has been used in our prior research with trans women [27]. Social cohesion is the level of solidarity within the community and was measured using Grover et al.’s [19] nine item scale (Cronbach’s alpha = 0.83) adapted for use with trans women and including items like “You can count on other trans women in your group of friends if you need to borrow money.” Consistent with the cut-offs used by Grover et al., responses to items in the scale were summed and then categorized into three social cohesion levels: low (or bottom 25^th^ percentile of scores), medium (or middle 50^th^ percentile of scores) or high (top 75^th^ percentile of scores). One item was used to measure acceptance in Nepali society. One measured whether or not trans women felt that Nepali society was not accepting of them as trans people. Specifically, we asked, “Are trans women an accepted part of Nepali society?” A 15-item version of the social participation index from van Brakel et al. [28] was used to measure perceived problems in socio-economic areas of life that are key to one’s survival. Items include questions like, “Do you have equal opportunity as peers to find work?” and “Do you contribute to the household economically in a similar way to your peers?” This measure has been field tested in Nepal [29] and has high internal consistency (Cronbach’s alpha) of 0.96. For the purposes of this analysis, social participation was dichotomized at its median score and categorized as high or low.

### Data analysis

Analyses were restricted to participants who lab-tested HIV negative (173/200–86.5%). From there, frequencies were calculated for socio-demographic characteristics and sex work status. The distribution of healthcare access, HIV testing behaviors, reasons for not getting tested for HIV, HIV knowledge, HIV stigma, anti-trans stigma, and stigma in healthcare among HIV negative trans women are described. Next, frequencies and means were computed for variables capturing social support, social cohesion, social acceptance of trans people in Nepal, and social participation.

Finally, tests for bivariable associations were conducted between negative indications of HIV testing with socio-demographics, HIV knowledge, HIV stigma, social support, social cohesion, social acceptance, social participation, anti-trans stigma, and healthcare stigma among HIV-negative participants. Poisson binomial regression models with robust variance were constructed to estimate prevalence ratios for each of the following outcomes: not testing for HIV in the last 3 months or not testing for HIV in the last 6 months. Given the relatively small sample size and exploratory nature of the analyses, we did not include any variables for adjustment. Confidence intervals that did not contain a prevalence ratio of 1 were considered statistically significant.

All descriptive and bivariable analyses were performed using STATA v.17. Our study received human subjects review and approval from the University of California, San Francisco, and the Nepal Health Research Council.

## Results

Socio-demographic characteristics and sex work engagement are presented in Table 1 for those who lab-tested HIV-negative (n = 173). About two-thirds of participants identified as trans women (110/173 = 63.6%), and the remaining third as *Tesoro Lingi* or another gender (63/173 = 36.4%). Age was uniformly distributed, and most participants completed grade school (89/173 = 51.4%), rented a room in a house or apartment (122/173 = 70.5%), and were employed full-time (123/173 = 71.1%). Sixty-eight participants (39.3%) reported monthly incomes of 12,000 NRS or less. Most participants reported lifetime (127/173 = 73.4%) and current sex work (101/173 = 58.4%).

**Table 1:** Socio-demographic characteristics and sex work status of HIV-negative trans women, Tesoro Lingi, or people with other genders in Nepal, 2019.

	n	%
*Total*	173	86.5%
*Socio-demographics*		
Gender		
Trans woman	110	63.6%
Tesoro Lingi or other^1^	63	36.4%
Age		
18-24 years	46	26.6%
24-34 years	56	32.4%
35+ years	71	41.0%
Education		
No formal education	23	13.3%
Grade school	89	51.4%
Secondary school graduation and above	61	35.3%
Current housing situation		
Own your own house	34	19.7%
Rent a house or apartment	13	7.5%
Rent a room in a house or apartment	122	70.5%
Living permanently with parents	3	1.7%
Living with parents until married	0	0.0%
Temporarily staying with parents	1	0.6%
Temporarily staying with friends or family	0	0.0%
Homeless/shelter	0	0.0%
Other	0	0.0%
Current employment		
Full-time^2^	123	71.1%
Part-time	21	12.1%
Student	13	7.5%
Unemployed	13	7.5%
Retired	3	1.7%
Current monthly income		
< 8,000 NRS	15	8.7%
8,000-12,000 NRS	53	30.6%
12,000-16,000 NRS	21	12.1%
16,000-20,000 NRS	39	22.5%
>20,000 NRS	34	19.7%
Missing	11	6.4%
*Sex work*		
Lifetime sex work	127	73.4%
Current sex work	101	58.4%

^1^ “Other” gender identity included gender queer (n = 1), and queer (n = 1).

^2^ Fulltime employment is inclusive of sex work.

Table 2 displays healthcare engagement and HIV testing behaviors, reasons for not getting tested, and HIV knowledge for HIV-negative trans women, Tesoro Lingi, and people with other genders in Nepal. While almost all participants had medical care access (170/173 = 98.3%), only about one in three (64/173 = 37.0%) were able to access gender affirming care.

**Table 2:** Healthcare access, HIV testing behaviors, reasons for not getting tested, and HIV knowledge reported by HIV-negative trans women, Tesoro Lingi, and people with other genders in Nepal, 2019 (n = 173).

	n	%
*Total*	173	100.0%
*Healthcare access*		
Have medical care in the city	170	98.3%
Have a doctor, nurse, or clinic where trans health care is obtained	64	37.0%
*Ever tested for HIV before*	157	90.8%
Where tested last (n = 157)		
Drop-in center	134	85.4%
Government hospital	14	8.9%
Private clinic	9	5.7%
Tested within last 3 months (n = 157)	84	53.5%
Tested within last 6 months (n = 157)	129	82.2%
Interested in home HIV testing (n = 157)	126	80.3%
*Reasons for not getting tested in the last 6 months (n = 44)*		
Thought at low risk for HIV	18	40.9%
Do not know where to get tested	6	13.6%
Afraid of receiving positive test result	10	22.7%
Think the test site counselor will tell others about HIV status	2	4.5%
Afraid treatment is not available	1	2.3%
Afraid of not being able to pay for treatment	1	2.3%
Afraid of needles	1	2.3%
Cannot afford to pay for testing	0	0.0%
Feel unwelcome at testing sites due to gender	0	0.0%
People will think participant is HIV positive if going to an HIV test site	0	0.0%
Other^1^	7	15.9%
*HIV knowledge*		
Composite score (range: 0-18), mean and SD	14.5	2.5
Belief that one can contract HIV from oral sex	117	67.6%

^1^ ‘Other’ responses included not having time in general and because of work and don’t know.

Most participants had received a HIV test prior to the study (157/173 = 90.8%). Most prior HIV tests were done at drop-in centers (134/157 = 85.4%), and about half of those reporting prior HIV tests had completed them within the last 3 months (84/157 = 53.5%). Moreover, most participants who received prior HIV testing reported interest in home HIV testing (126/157 = 80.3%). Forty-four participants received their most recent HIV test more than 6 months ago. Of those 44, low perceived HIV risk (18/44 = 40.9%) and fear of a positive HIV test result (10/44 = 22.7%) were the most cited reasons for not recently being tested for HIV. The mean HIV knowledge score was 14.5 (SD = 2.5, range = 0–18).

There was a mix of types of responses with societal stigma measures being high along with high regard for the rights of people living with HIV. Over 90% of participants believed that most people in Nepal would discriminate against people with HIV or believed that most people in Nepal would think that those with HIV got what they deserved. Conversely, most of the sample also believed that most people in Nepal would support the rights of someone with HIV (131/173 = 75.7%) or be friends with someone with HIV (117/173 = 67.6%) (Table 3). There was also a high prevalence of anti-trans and healthcare-related stigma reported by HIV-negative participants in Nepal. Most participants hid their gender identity from their family (161/173 = 93.1%) or were arrested for being trans (89/173 = 51.4%). Further, about one in five of these participants educated their doctor about transgender health (36/173 = 20.8%), and almost one in two experienced discrimination in medical care due to their gender identity (84/173 = 48.6%) (Table 3).

**Table 3:** Stigmas, social support, cohesion, acceptance, and participation among HIV-negative trans women, Tesoro Lingi, and other gender people in Nepal, 2019–2020 (n = 173).

	n	%
*Total*	173	100.0%
*HIV Stigma*		
Most people in Nepal would discriminate against someone with HIV	163	94.2%
Most people in Nepal would support the rights of someone living with HIV	131	75.7%
Most people in Nepal would be friends with someone with HIV	117	67.6%
Most people in Nepal think those with HIV got what they deserve	167	96.5%
*Anti-trans stigma*		
Hid gender identity from family in the last year	161	93.1%
Arrested for being trans	89	51.4%
*Stigma in Healthcare*		
Had to educate doctor about transgender health	36	20.8%
Experienced maltreatment in medical care because of gender identity	84	48.6%
Mistreatment from a clinic staff person because of gender identity	14	8.1%
*Social support*		
Composite score (range: 1-4), mean and SD	3.4	0.4
Low/medium perceived support (25-36)	49	28.3%
High perceived support (37-48)	122	70.5%
*Social cohesion*		
Composite score (range: 0-36), mean and SD	20.3	5.7
Low (0-17)	43	24.9%
Medium (18-23)	72	41.6%
High (24-36)	58	33.5%
*Social acceptance of trans people in Nepal*		
Yes	55	31.8%
No	114	65.9%
*Social participation*		
High	96	55.5%
Low	77	44.5%

Social support, cohesion, acceptance, and participation among participants in Nepal are presented in Table 3. Mean social support was 3.4 out of a total score of 4.0, with 122 (70.5%) HIV-negative participants citing high social support. The average social cohesion score was 20.3 out of 36, with one quarter reporting low social cohesion (43/173 = 24.9%), almost half reporting medium social cohesion (72/173 = 41.6%), and about one third reporting high social cohesion (58/173 = 33.5%). Less than one-third of participants felt that trans women were accepted by Nepali society (55/173 = 31.8%). Just over half had high social participation in Nepali society (96/173 = 55.5%).

Table 4 shows bivariable associations between HIV testing and socio-demographics, HIV knowledge, HIV stigma, social support, social cohesion, social acceptance, social participation, anti-trans stigma, and healthcare stigma among HIV-negative participants. Participants who reported current sex work had lower prevalence of not testing for HIV in the last 3 months compared to those who did not report current sex work (prevalence ratio, PR = 0.54, 95% confidence interval, 95%CI = 0.32–0.92, p = 0.02). Every one-unit increase in social cohesion was associated with 1.05 times the prevalence of not testing for HIV in the last 3 months (95%CI = 1.01–1.09, p-value = 0.02). Those with higher HIV knowledge had lower prevalence of not being tested for HIV in the last 6 months (prevalence ratio, PR = 0.89, 95% confidence interval, 95%CI = 0.82–0.97, p <0.01). Bivariable comparisons for other characteristics did not reach statistical significance.

**Table 4:** Bivariable associations between negative indications of HIV testing engagement and socio-demographics, HIV knowledge, HIV stigma, social support, social cohesion, social acceptance, social participation, anti-trans stigma, and healthcare stigma among HIV-negative trans women, Tesoro Lingi, and people with other genders in Nepal (n = 173), 2019–2020.

	Not tested for HIV, last 3 months			Not tested for HIV, last 6 months		
	PR^a^	95% CI^b^	p-value	PR	95% CI	p-value
*Socio-demographics*						
Education						
No formal education or grade school	Ref^c^			Ref		
Graduation and above	1.02	(0.65-1.59)	0.93	1.15	(0.63-2.12)	0.64
Sex work						
Current	0.54	(0.32-0.92)	0.02	0.54	(0.25-1.15)	0.11
Lifetime	1.02	(0.63-1.66)	0.93	0.86	(0.45-1.65)	0.66
*HIV Knowledge*						
Composite score (range: 0-18)	0.94	(0.88-1.01)	0.10	0.89	(0.82-0.97)	< 0.01
Belief that one can contract HIV from oral sex	0.67	(0.43-1.06)	0.09	0.78	(0.40-1.50)	0.45
*HIV Stigma*						
Most people in Nepal would discriminate against someone with HIV	0.80	(0.35-1.82)	0.59	0.61	(0.22-1.71)	0.35
Most people in Nepal would support the rights of someone living with HIV	0.85	(0.53-1.37)	0.51	0.76	(0.40-1.46)	0.42
Most people in Nepal would be friends with someone with HIV	0.78	(0.51-1.21)	0.26	0.63	(0.35-1.14)	0.13
Most people in Nepal think those with HIV got what they deserve	0.72	(0.26-1.96)	0.52	0.75	(0.18-3.12)	0.70
*Social support*						
Composite score (range: 1-4)	1.08	(0.65-1.78)	0.77	1.01	(0.51-2.01)	0.97
*Social cohesion*						
Composite score (range: 0-36)	1.05	(1.01-1.09)	0.02	1.01	0.96-1.07	0.63
*Social acceptance of trans people in Nepal*						
Yes	Ref			Ref		
No	1.06	(0.67-1.68)	0.81	0.90	(0.48-1.67)	0.74
*Social participation*						
High	Ref			Ref		
Low	1.19	(0.77-1.82)	0.43	1.50	(0.83-2.71)	0.18
*Anti-trans stigma*						
Hid gender identity from family in the last year	0.88	(0.38-2.01)	0.76	0.67	(0.24-1.86)	0.44
Arrested for being trans	0.90	(0.59-1.38)	0.63	0.59	(0.32-1.09)	0.09
*Stigma in Healthcare*						
Had to educate doctor about trans health	0.90	(0.52-1.54)	0.69	0.72	(0.32-1.61)	0.43
Experienced maltreatment in medical care because of gender identity	0.70	(0.45-1.10)	0.12	0.56	(0.30-1.04)	0.07
Mistreatment from a clinic staff person because of gender identity	1.20	(0.58-2.50)	0.62	1.13	(0.40-3.15)	0.82

Note

^a^ Prevalence ratio.

^b^ Confidence interval.

^c^ Reference group.

## Discussion

Trans women in our study faced significant and unique barriers to HIV testing in Nepal that may be influenced by stigma. Despite targeted HIV outreach efforts to key populations in Nepal [30], 10% of trans women in our study had never been tested for HIV, and almost half had not been recently tested. Testing prevalence among trans women in our study was higher than in Nepal overall, which data from 2016 estimates is between 10–20% of cisgender men and women [31]. However, overall testing prevalence and recency of testing was low among trans women in Nepal and occurred within the context of high stigma creating many risk factors for HIV and barriers to prevention.

Overall low recent HIV testing among trans women may be explained, in part, by the need for social connection to buffer from stigma in a high HIV stigma environment. The majority of trans women not living with HIV in our study had medium to high social cohesion and social support. Participants also held strong HIV stigma beliefs. Almost all participants in our study believed that most people in Nepal would discriminate against someone living with HIV, and that people in Nepal thought that those with HIV got what they deserve. Notably, almost no trans women were concerned about HIV treatment access if they were to test positive, so fear of treatment availability was not a concern as it may be in other low-income country settings [32]. It could be the case that anticipated HIV stigma in this group of socially connected trans women may prevent them from wanting to know if they are living with HIV. Said differently, the risk of losing connection to the social network may too high for trans women to risk potential rejection from testing positive for HIV. Future work to understand the dynamics within trans women’s social networks and HIV stigma views within the community are needed to further explore this phenomenon.

Support for this connection exists in our bivariable analysis wherein we found that higher social cohesion was associated with significantly lower likelihood to have been tested for HIV recently. We also found that fear of getting a positive HIV result was listed as one of the main barriers to HIV testing, and this fear may be heightened by HIV stigma. HIV knowledge was also significantly associated with lower prevalence of not HIV testing recently. Research has found that trans women in other countries may avoid regular HIV testing because they anticipate social costs associated with testing positive for HIV [33–35]. Fear of losing social connections due to HIV stigma, either when being seen getting tested for HIV or if they test positive, may be what resulted in relatively low recent HIV testing among trans women in our study [36].

We also found that sex work was significantly associated with recent HIV testing in bivariable analysis. Globally, one in ten sex workers are living with HIV [37]. Trans women in our study may have been aware of their elevated HIV risk and engaged in HIV testing to maintain their health. More than half of trans women in our study were currently sex workers, which is high and consistent with the high representation in sex work among trans women due to structural barriers from anti-trans discrimination [37]. In Kathmandu, there is at least one organization whose specific focus is health and HIV prevention outreach to serve sex workers. Participants in our study may have also benefitted from these expert outreach efforts.

Several potential avenues for intervention can be gleaned from this study. Since trans women who do sex work were significantly more likely to have tested for HIV recently, engaging trans women who do sex work for insight into effective HIV testing interventions may be an important strategy to improve HIV testing among trans women overall in Nepal. In addition, efforts to improve HIV knowledge and awareness of HIV testing availability among trans women may yield increases in frequency of HIV testing. HIV self-testing may also be an important intervention for trans women in Nepal that can help increase HIV testing frequency and mitigate stigma. Local research found almost universal willingness to try HIV self-testing among trans women [38]. This finding is consistent with other data from 2018 studying the uptake and acceptability of self-testing for HIV among trans women and MSM in Nepal in 2018. In that study, 100% of trans women offered a HIV self-test and given an option to have assisted self-testing rather than taking the test unassisted were willing to self-test for HIV [38]. Trans women who can test at home have less risk of their peers finding that they have tested positive and can get support to determine the best way to maintain social connection when they decide to disclose their HIV status [39]. Interventions like HIV self-testing that address stigma may also be needed for successful implementation of novel interventions like PrEP, which was not available at the time of this study.

The primary limitation for this analysis was the study sample size which may have limited our ability to detect relationships between these intricate measures and HIV testing behaviors and prevented us from conducting adjusted analyses. This exploratory analysis also elucidated the need for more measures of stigma that expand each type of stigma. Because the study was cross-sectional, we also were not able to examine the relationships between stigma and HIV testing over time, which if we could have done so, may have elucidated how knowledge and social relationships impact HIV prevention behaviors. Also, the measures did not examine the strength and types of social cohesion and support, therefore, we do not have in depth data on the types of support most accessed and valued by trans women.

Despite limitations, we believe these exploratory findings support the need for further examination into the importance and impact of social relationships on HIV care and prevention engagement among marginalized populations like trans women in low-income settings. Specifically, analyses that focus on understanding the social costs to testing positive for trans women are needed, as are interventions that address HIV stigma within marginalized communities and in society. Altogether, this analysis also points to a need for novel interventions that address stigma in efforts to improve regular engagement in HIV prevention among trans women in Nepal. As scale of PrEP begins in Nepal, efforts to address stigma are ever more pressing to ensure access and utilization among trans women and other marginalized populations effected by the HIV epidemic. Furthermore, while data are not available for the regional and country-wide impact of the Covid-19 pandemic on HIV testing in Nepal post implementation of this study, we anticipate major impacts due to intermittent shelter in place ordinances, multiple waves of the epidemic and supply chain issues. And we know that globally HIV testing rates declined for the first time in 20 years as a result of the pandemic [40]. Thus, efforts to increase HIV-related health seeking behaviors are also needed to address new barriers from the global COVID-19 pandemic among trans women in Nepal.
